# Bias in vaccine effectiveness studies of clinically severe outcomes that are measured with low specificity: the example of COVID-19-related hospitalisation

**DOI:** 10.2807/1560-7917.ES.2024.29.7.2300259

**Published:** 2024-02-15

**Authors:** Christian Holm Hansen

**Affiliations:** 1 Department of Infectious Disease Epidemiology and Prevention, Statens Serum Institut, Copenhagen, Denmark; 2 MRC International Statistics and Epidemiology Group, London School of Hygiene and Tropical Medicine, London, United Kingdom

**Keywords:** COVID-19, vaccine effectiveness, sensitivity and specificity, bias, methods

## Abstract

Many vaccine effectiveness (VE) analyses of severe disease outcomes such as hospitalisation and death include ‘false’ cases that are not actually caused by the infection or disease under study. While the inclusion of such false cases inflate outcome rates in both vaccinated and unvaccinated populations, it is less obvious how they affect estimates of VE. Illustrating the main points through simple examples, this article shows how VE is underestimated when false cases are included as outcomes. Depending how the outcome indicator is defined, estimates of VE against severe disease outcomes, whose definition allows for the inclusion of false cases, will be biased downwards and may in certain circumstances approximate the same level as the VE against infection. The bias is particularly pronounced for vaccines that offer high levels of protection against severe disease outcomes but poor protection against infection. Analysing outcomes that are measured with low sensitivity generally does not cause bias in VE studies; defining outcome indicators that minimise the number of false cases rather than the number of missed cases is preferable in VE studies.

## Introduction

The severe acute respiratory syndrome coronavirus 2 (SARS-CoV-2) Omicron variant (Phylogenetic Assignment of Named Global Outbreak Lineages (Pangolin) designation B.1.1.529), which appeared towards the end of 2021, was generally associated with milder disease than previous SARS-CoV-2 variants but also with higher transmission rates [1-4]. At the height of the Omicron wave, a large number of hospital patients therefore happened to be SARS-CoV-2-positive despite being hospitalised for reasons unrelated to COVID-19 [5]. Analyses that attributed such hospitalisations to COVID-19, simply because of the co-occurrence in time with a SARS-CoV-2 infection, exaggerated the number of hospitalisations supposedly due to COVID-19 [6]. But how does such outcome misclassification affect estimates of vaccine effectiveness (VE)?

Vaccine effectiveness is essentially a measure of the protective immunological effect induced by a vaccine against a certain outcome of interest such as infection, disease, hospitalisation or death. It is defined as the ratio of the case rate in vaccinated people to that in unvaccinated people. Valid estimates in VE studies are important as such studies help inform public health decisions. Observational VE studies are routinely used to monitor the protective effect in a population, or segments of a population, of new and established vaccines, for example those against seasonal diseases such as influenza and COVID-19, but they are also used in more unusual outbreaks as seen recently with studies of vaccine protection against mpox [7-9].

In this article, we investigate how outcome misclassification affects estimates in VE studies of severe outcomes. The first section presents the theoretical background and is followed by a section illustrating the main points through six simple scenarios using the example of VE against COVID-19-related hospitalisation and finally some suggestions for assessing the magnitude of the bias based on a few assumptions.

## Theoretical background

Define VE against infection with a particular pathogen as *VE_inf_ = 1 − (π_i1_/π_i0_)* where *π_i1_* and *π_i0_* are the infection rates in the vaccinated and unvaccinated population respectively. 

If the outcome of interest is a disease outcome caused by the infection, e.g. hospitalisation, rather than just infection itself, the quantity we seek to estimate is

$${VE}_{hos}=1-\frac{\pi_{i1}\times \pi_{h1}}{\pi_{i0}\times \pi_{h0}}\;\;(Formula1)$$

where *π_h1_* and *π_h0_* denote the probability that an infection in a vaccinated and unvaccinated person, respectively, will lead to the outcome. For argument’s sake, but without loss of generality, this outcome is taken to be hospitalisation from this point onwards.

In studies using case definitions with low specificity, however, the measured outcome rates will be inflated by the inclusion of misclassified cases. For example, if individuals hospitalised for other reasons also have an ‘incidental’ infection with the pathogen studied and are erroneously included as cases in the analysis, the quantity actually being estimated is

$${VE'}_{hos}=1-\frac{\pi_{i1}\times \pi_{h1}+\pi_{i1}\left(1-\pi_{h1}\right)\times \pi_{f}}{\pi_{i0}\times \pi_{h0}+\pi_{i0}\left(1-\pi_{h0}\right)\times \pi_{f}}\;\;\;\;(Formula\;2)$$

Here *π_f_* is the probability that a randomly selected person from the population is hospitalised for reasons unrelated to the studied infection and is assumed independent of vaccination status and infection.

In the numerator of the ratio measure in Formula 2, note that the term *π_i1_* × *π_h1_* is retained from Formula 1 and represents the proportion of the vaccinated population that is hospitalised due to the infection. The additional term, *π_i1_*(1 − *π_h1_*) × *π_f_*, is the proportion among the vaccinated population that is infected and hospitalised due to other causes. The denominator is of the same form and relates to the unvaccinated population.

We can rewrite Formulae 1 and 2 in terms of relative rates (RR),

$${RR}_{hos}\;=\;1-{VE}_{hos}\;=\;(1-{VE}_{inf})\frac{\pi_{h1}}{\pi_{h0}}\;\;(Formula\;3)\;$$

$${RR'}_{hos}\;=\;1-{VE^{'}}_{hos}\;=\;\left(1-{VE}_{inf}\right)\frac{\pi_{h1}+\left(1-\pi_{h1}\right)\times \pi_{f}}{\pi_{h0}+\left(1-\pi_{h0}\right)\times \pi_{f}}\;\;(Formula\;4)$$

Comparing the expressions in Formulae 3 and 4, and assuming that *π_h1_* ≤ *π_h0_*, it can be seen that *RR_hos_* ≤ *RR’_hos_*, or equivalently that *VE’_hos_* ≤ *VE_hos_*, since


$$\frac{\pi_{h1}}{\pi_{h0}}\leq \frac{\pi_{h1}+(1-\pi_{h1})\times \pi_{f}}{\pi_{h0}+(1-\pi_{h0})\times \pi_{f}}$$

It can also be seen from Formula 4 that *VE’_hos_* tends towards *VE_inf_* as *π_f_* approaches 1. We therefore have *VE_hos_* ≥ *VE’_hos_* ≥ *VE_inf_*.

By introducing a bias correction factor *c* we can express *RR_hos_* as a function of *RR’_hos_*

*RR_hos_* = *c.RR’_hos_* (Formula 5)

$$where\;c=\frac{(1-VEinf)\frac{\pi_{h1}}{\pi_{h0}}}{(1-VEinf)\frac{\pi_{h1}+(1-\pi_{h1})\times \pi_{f}}{\pi_{h0}+(1-\pi_{h0})\times \pi_{f}}}=\frac{\pi_{h1}}{\pi_{h0}}\times \frac{\pi_{h0}+(1-\pi_{h0})\times \pi_{f}}{\pi_{h1}+(1-\pi_{h1})\times \pi_{f}}$$

is a value ranging from 1 to *π_h1_*/*π_h0_* as *π_f_* ranges from 0 to 1. Note that the bias is greatest when *π_h1_* is a lot smaller than *π_h0_* and when *π_f_* is large relative to *π_h0_* and *π_h1_*. Meanwhile there is no bias (*c = 1*) when *π_h1_* = *π_h0,_* since *VE_hos_* = *VE_inf_* at that point. This would be the case for a vaccine which may protect against infection but does not provide any further protection against hospitalisation once infection has occurred. Note also that there is no relationship between the size of the bias, *c,* and the infection rates as expressed through *π_i0_* and *π_i1_*.

The relationship in Formula 5 can be rewritten as *VE_hos_* = *1 − c(1 − VE’_hos_)*.

## Illustrating examples

In the following, the results derived above are demonstrated through a number of scenarios using the example of VE against COVID-19-related hospitalisation. The first three scenarios present examples of a vaccine with a relatively high level of effectiveness against infection, 80%, while the VE against infection in Scenarios 4–6 is only 20%. The scenarios are hypothetical and have been selected to illustrate the relationship between the VE as estimated in a study and the parameters introduced above.

### Scenario 1: Base case scenario

In Scenario 1 (see Table), the proportion of the population who are positive for SARS-CoV-2 is 10% among unvaccinated (*π_i0_* = 0.10) and 2% among vaccinated people (*π_i1_* = 0.02), meaning that VE against infection is 80%. Once infected, vaccinated individuals are less likely than unvaccinated individuals to require hospitalisation as only 0.5% of vaccinated cases are hospitalised because of COVID-19 (*π_h1_* = 0.005) compared with 2% of unvaccinated cases (*π_h0_* = 0.02). The true RR of hospitalisation for COVID-19 in the population is therefore (0.02 × 0.005) / (0.10 × 0.02) = 0.05 resulting in a VE against COVID-19 hospitalisation of 95%. The *estimated* VE of 87.4% is lower, however, due to contamination from individuals with an ‘incidental’ SARS-CoV-2 diagnosis, i.e. individuals who happen to be infected but who are actually hospitalised for other reasons. Two per cent of the population under study, whether vaccinated or unvaccinated, is in hospital for other reasons (*π_f_* = 0.02). The observed COVID-19 hospitalisation rates are consequently inflated in both the vaccinated and unvaccinated group by an amount that is equal to 2% of the total infections observed in the two groups. This forces the numerator and denominator of the RR closer together and pushes down the VE estimate.

**Table:** Six scenarios illustrating bias in estimated vaccine effectiveness against hospitalisation for COVID-19 in studies with low specificity of outcome

	Total population	Infection rates, π_i_	Number of infected individuals	VE against infection, 1−(π_i1_/π_i0_) × 100%	Rates of hospitalisation for COVID-19 among infected, π_h_	Rate of hospitalisation for other reasons, π_f_	Hospitalised individuals with a SARS-CoV-2 infection		True VE against hospitalisation for COVID-19	Observed VE against hospitalisation for COVID-19
							Number of hospitalisations that are because of COVID-19	Number of hospitalisations for reasons other than COVID-19		
Scenario 1: Base case scenario										
Vaccinated	1,000,000	π_i1_ = 0.02	20,000	80%	π_h1_ = 0.005	0.02	100	398	1−(100/2,000): 95%	1−(498/3,960): 87.4%
Unvaccinated	1,000,000	π_i0_ = 0.10	100,000		π_h0_ = 0.020		2,000	1,960		
Scenario 2: 10 times lower infection rates										
Vaccinated	1,000,000	π_i1_ = 0.002	2,000	80%	π_h1_ = 0.005	0.02	10	40	1−(10/ 200): 95%	1−(50/ 396): 87.4%
Unvaccinated	1,000,000	π_i0_ = 0.010	10,000		π_h0_ = 0.020		200	196		
Scenario 3: 10 times less severe disease										
Vaccinated	1,000,000	π_i1_ = 0.02	20,000	80%	π_h1_ = 0.0005	0.02	10	400	1−(10/ 200): 95%	1−(410/2,196): 81.3%
Unvaccinated	1,000,000	π_i0_ = 0.10	100,000		π_h0_ = 0.0020		200	1,996		
Scenario 4: Poor vaccine effectiveness against infection										
Vaccinated	1,000,000	π_i1_ = 0.08	80,000	20%	π_h1_ = 0.005	0.02	400	1,592	1−(400/2,000): 80%	1−(1,992/3,960): 49.7%
Unvaccinated	1,000,000	π_i0_ = 0.10	100,000		π_h0_ = 0.020		2,000	1,960		
Scenario 5: Poor vaccine effectiveness against infection; good vaccine protection once infected										
Vaccinated	1,000,000	π_i1_ = 0.08	80,000	20%	π_h1_ = 0.002	0.02	160	1,597	1−(160/2,000): 92%	1−(1,757/3,960): 55.6%
Unvaccinated	1,000,000	π_i0_ = 0.10	100,000		π_h0_ = 0.020		2,000	1,960		
Scenario 6: Poor vaccine effectiveness against infection; good vaccine protection once infected; low rates of hospitalisation for other reasons										
Vaccinated	1,000,000	π_i1_ = 0.08	80,000	20%	π_h1_ = 0.002	0.001	160	80	1−(160/2,000): 92%	1−(240/2,098): 88.6%
Unvaccinated	1,000,000	π_i0_ = 0.10	100,000		π_h0_ = 0.020		2,000	98		

SARS-CoV-2: severe acute respiratory syndrome coronavirus 2; VE: vaccine effectiveness.

The infection rate is 10% among the unvaccinated population except in Scenario 2 where it is 1%. The risk of hospitalisation due to COVID-19 following an infection is 2% among the unvaccinated population except in Scenario 3 where it is 0.2%. Except in Scenario 6 where it is 0.1%, the risk of hospitalisation in the population among those not hospitalised for reasons related to COVID-19 is 2%, e.g. (20,000–100) × 0.02 = 398 in Scenario 1.

### Scenario 2: 10 times lower infection rates

As was established above, the incidence rate of infection in the population, reflecting the contagiousness of the pathogen, does not impact the magnitude of this bias (the bias correction factor *c* is independent of *π_i0_* and *π_i1_*). This is illustrated in Scenario 2 where the infection rate is 10 times smaller in both the vaccinated and unvaccinated population (*π_i0_* = 0.01, *π_i1_* = 0.002) compared with Scenario 1 but the estimated VE remains unchanged.

### Scenario 3: 10 times less severe disease

In Scenario 3, the disease is milder as the risk that an infection will lead to hospitalisation is 10 times less that in Scenario 1 (*π_h0_* = 0.002, *π_h1_* = 0.0005). Relative to the many misclassified cases with incidental infection, the relevant contrast between vaccinated and unvaccinated cases that are actually hospitalised for COVID-19 is diluted considerably. If the number of hospitalisations with incidental infections is large relative to those actually hospitalised due to the infection under study, the observed hospitalisation rate ratio, which in Scenario 3 is (400 + 10) / (1,996 + 200) = 410/2,196, will approach the rate ratio for infections among vaccinated vs unvaccinated (20,000/100,000). Consequently, as was established above, we see in Scenario 3 that the estimated VE against hospitalisation approaches the level of VE against infection.

### Scenario 4: Poor vaccine effectiveness against infection

When VE for infection is high, as in Scenarios 1–3, the absolute scale of the bias may be limited. On the other hand, when VE for infection is low (e.g. 20% as in Scenario 4), estimates of VE against hospitalisation may tend towards similarly low levels despite good vaccine protection against hospitalisation once infected.

### Scenario 5: Poor vaccine effectiveness against infection; good vaccine protection once infected

The bias is particularly pronounced for vaccines that offer high levels of protection against hospitalisation despite poor protection against infection as was the case for many of the original (monovalent) COVID-19 vaccines during the Omicron era [10,11]. This is illustrated further in Scenario 5 where the vaccine still only protects 20% against infection but vaccinated SARS-CoV-2 cases are 10 times less likely to require hospitalisation than unvaccinated cases (*π_h1_*/*π_h0_* = 0.002/0.02 = 0.1). Here, VE against hospitalisation is estimated at just 55.6% when in fact it is 92%.

### Scenario 6: Poor vaccine effectiveness against infection; good vaccine protection once infected, low rates of hospitalisations for other reasons

Arguably most important for the magnitude of the bias is the rate of misclassified cases, *π_f_*, i.e. in this example, the proportion of people in hospital for reasons other than COVID-19. This is illustrated in Scenario 6, which is a repeat of Scenario 5 except only 0.1% of the total population under study (not hospitalised for COVID-19) is in hospital for other reasons (*π_f_* = 0.001). In this Scenario, the estimated VE of 88.6% is wrong by only a few percentage points. In many real-life scenarios, a lower *π_f_* such as in Scenario 6 may be more realistic, especially if the study is conducted in a general population of relatively good health.

## Bias correction

As explained above, the true (unbiased) VE against a severe disease outcome, such as hospitalisation, can be expressed as *VE_hos_ = 1 – c × (1 –VE’_hos_),* where *VE’_hos_* is the estimated VE and *c* is the bias correction factor


$$c=\frac{\pi_{h1}}{\pi_{h0}}\times \frac{\pi_{h0}+(1-\pi_{h0})\pi_{f}}{\pi_{h1}+\left(1-\pi_{h1}\right)\pi_{f}}$$

which is a function of the three parameters, *π_f_*, *π_h1_* and *π_h0_*. To illustrate, having observed *VE’_hos_ = 81.3%* as in Scenario 3, and assuming *π_f_* = 0.02, *π_h1_* = 0.0005 and *π_h0_* = 0.002, we can evaluate *c* as 0.2679 and the true VE as 1 – 0.2679 × (1 – 0.813) = 95%.

In practice *π_f_, π_h1_* and *π_h0_* will generally not be known but might be gauged from electronic health records or external studies so that it may still be possible to gain a sense of the level of underestimation that can be expected in particular scenarios and, by varying the parameters, suggest a range of plausible values within which the true (unbiased) VE is likely to be.

## Discussion

Generally, a low specificity of the outcome measure in clinical studies results in rates being overestimated in both treatment and control groups. Consequently, it is well established that low specificity attenuates the ratio of the measured rates causing the ratio to be closer to 1 than it truly is [12,13]. However, in studies of vaccine protection against severe outcomes, VE is typically derived from the product of two ratios, namely the ratio of infection rates and secondly, among those infected, the ratio of the rates of severe outcome. In many studies, such as those using PCR methods to detect infection, the problem of low specificity affects only the second ratio. Consequently, as illustrated in this article, the biased estimate is bounded by the level of VE against infection.

We have seen that the proportion of individuals admitted to hospital for unrelated reasons, *π_f_*, is an important factor for the size of the bias. In all the scenarios shown, and in the expression for *c*, it is assumed that *π_f_* is independent of vaccination status. A fundamental principle of VE studies is that the vaccinated and unvaccinated groups being compared do not differ systematically with respect to other risk factors (at least not after adjustment). This is necessary to ensure that the VE measure captures only the immunological effect of vaccination and not the effects of other exposure and disease predictors. It would probably be a sign that the health profiles are not comparable if *π_f_* differed between the two groups, and the resultant VE estimate would therefore also be biased due to confounding. Supplementary analyses with negative control exposures or outcomes are recommended in observational VE studies as a way to assess such bias [14,15]. Nonetheless, it is possible to adapt the expression for *c* to accommodate different *π_f_* rates, say *π_f1_* and *π_f0_* respectively, in the vaccinated and unvaccinated group; it can then be shown that the underestimation is even more pronounced if *π_f1_* > *π_f0_*, which would be the situation if, for example, people of poorer health were more likely to be vaccinated.

Observational studies to estimate VE may be designed in various ways. Study designs include retrospective cohort studies through analysis of routinely collected electronic health records, cross-sectional designs, prospective cohort studies, test-negative designs and other case–control studies. The biasing effects presented in this article, caused by outcome misclassification, apply equally across all these designs. Even randomised controlled trials (RCTs) are vulnerable to this type of outcome misclassification bias, although due to their budgets and rigour, RCTs often include more accurate outcome assessment methods than observational studies.

Unlike the problems caused by low specificity, low sensitivity of an outcome measure generally does not bias the ratio of the measured rates in the two groups [13]. It is therefore preferable for studies of VE to use outcomes that minimise numbers of misclassified cases (false cases) rather than numbers of missed cases.

Depending on data availability, minimising the number of misclassified cases may be achieved by defining more specific outcome measures based for example on primary diagnosis codes upon hospital admission, hospital procedures or death certificate information. Death as a specific disease outcome may also be defined with higher specificity, but possibly at the cost of lower sensitivity, by requiring certain accompanying hospital diagnoses or procedures. A number of studies have explored alternative severe outcome definitions in the context of VE [6,16,17].

Whether in the context of COVID-19, influenza or some other type of infection, studies of VE against severe disease outcomes such as hospitalisation and death should aim to use outcome measures that minimise inclusion of false cases to avoid underestimation of the effects of interest. Where this is not possible, consideration should be given to the magnitude of the resulting bias, for example by investigating likely scenarios for the three parameters that enter the expression for *c* above.

## Conclusions

Vaccine effectiveness against severe disease outcomes will generally be underestimated when incidental cases are included in the analysis. The potential error is greatest for vaccines that offer high levels of protection against severe disease but poor protection against infection. Outcomes with high specificity rather than high sensitivity are preferable in VE studies.
